# *In Vitro* Evaluation of a Soluble *Leishmania* Promastigote Surface Antigen as a Potential Vaccine Candidate against Human Leishmaniasis

**DOI:** 10.1371/journal.pone.0092708

**Published:** 2014-05-02

**Authors:** Rym Chamakh-Ayari, Rachel Bras-Gonçalves, Narges Bahi-Jaber, Elodie Petitdidier, Wafa Markikou-Ouni, Karim Aoun, Javier Moreno, Eugenia Carrillo, Poonam Salotra, Himanshu Kaushal, Narender Singh Negi, Jorge Arevalo, Francesca Falconi-Agapito, Angela Privat, Maria Cruz, Julie Pagniez, Gérard-Marie Papierok, Faten Bel Haj Rhouma, Pilar Torres, Jean-Loup Lemesre, Mehdi Chenik, Amel Meddeb-Garnaoui

**Affiliations:** 1 Laboratory of Medical Parasitology, Biotechnology and Biomolecules, LR11-IPT-06, Institut Pasteur de Tunis, Tunis, Tunisia; 2 Institut de Recherche pour le Développement, UMR177 IRD/CIRAD «INTERTRYP», Montpellier, France; 3 Laboratory of Transmission, Control and Immunobiology of Infection, LR11-IPT-02, Institut Pasteur de Tunis, Tunis, Tunisie; 4 UPSP EGEAL Institut Polytechnique LaSalle Beauvais, Beauvais, France; 5 WHO Collaborating Center for Leishmaniasis, Centro Nacional de Microbiologia, Instituto de Salud Carlos III, Madrid, Spain; 6 National Institute of Pathology (ICMR), Safdarjung Hospital Campus, New Delhi, India; 7 Instituto de Medicina Tropical “Alexander von Humboldt”, Universidad Peruana Cayetano Heredia (UPCH), Lima, Peru; 8 BVT-VIRBAC, La Seyne sur Mer, France; 9 Centro de Transfusión de la Comunidad de Madrid, Madrid, Spain; INRS - Institut Armand Frappier, Canada

## Abstract

PSA (Promastigote Surface Antigen) belongs to a family of membrane-bound and secreted proteins present in several *Leishmania* (*L*.) species. PSA is recognized by human Th1 cells and provides a high degree of protection in vaccinated mice. We evaluated humoral and cellular immune responses induced by a *L. amazonensis* PSA protein (*La*PSA-38S) produced in a *L. tarentolae* expression system. This was done in individuals cured of cutaneous leishmaniasis due to *L. major* (CCLm) or *L. braziliensis* (CCLb) or visceral leishmaniasis due to *L. donovani* (CVLd) and in healthy individuals. Healthy individuals were subdivided into immune (HHR-*Lm* and HHR-*Li*: Healthy High Responders living in an endemic area for *L. major* or *L. infantum* infection) or non immune/naive individuals (HLR: Healthy Low Responders), depending on whether they produce high or low levels of IFN-γ in response to *Leishmania* soluble antigen. Low levels of total IgG antibodies to *La*PSA-38S were detected in sera from the studied groups. Interestingly, *La*PSA-38S induced specific and significant levels of IFN-γ, granzyme B and IL-10 in CCLm, HHR-*Lm* and HHR-*Li* groups, with HHR-*Li* group producing TNF-α in more. No significant cytokine response was observed in individuals immune to *L. braziliensis* or *L. donovani* infection. Phenotypic analysis showed a significant increase in CD4+ T cells producing IFN-γ after *La*PSA-38S stimulation, in CCLm. A high positive correlation was observed between the percentage of IFN-γ-producing CD4+ T cells and the released IFN-γ. We showed that the *La*PSA-38S protein was able to induce a mixed Th1 and Th2/Treg cytokine response in individuals with immunity to *L. major* or *L. infantum* infection indicating that it may be exploited as a vaccine candidate. We also showed, to our knowledge for the first time, the capacity of *Leishmania* PSA protein to induce granzyme B production in humans with immunity to *L. major* and *L. infantum* infection.

## Introduction

*Leishmania* (*L.*) are intracellular protozoa that cause a wide spectrum of human diseases, ranging from self-healing cutaneous to lethal visceral leishmaniasis. Cutaneous leishmaniasis (CL) is widely distributed in the Americas, North Africa, the Middle East and Central Asia, causing considerable morbidity [1]. In Tunisia, CL is zoonotic and is mainly due to *L*. *major* [2]. In Peru, *L. braziliensis* is responsible for nearly all the zoonotic CL cases [3]. Anthroponotic Visceral Leishmaniasis (VL) due to *L. donovani* is mainly distributed in the Indian subcontinent with over 300 000 annual cases [4], whereas zoonotic VL caused by *L. infantum* is present mainly in Mediterranean basin, Middle east, central Asia and Brazil with up to 50 000 annual cases [5]–[7]. Treatment of leishmaniasis mainly relies on chemotherapy and the control of the disease is challenged by serious side effects of existing drugs and the widespread emergence of drug resistant strains. Several lines of evidence are in favor of the feasibility of a vaccine in humans. Healing is generally associated with the development of a life-long immunity to re-infection. Moreover, a large majority of immuno-competent exposed individuals develop asymptomatic or subclinical infections rather than a severe form of leishmaniasis (symptomatic) and acquires a robust and durable immunity to reinfection. Resistance to infection is based on a Th1 dominant response with interferon (IFN)-γ production resulting in macrophage activation and parasite killing [8]–[10]. In healed individuals, it has been shown that recovery and resistance to re-infection also correlate with the development of antigen-specific Th1 cell responses and IFN-γ production [11]–[14]. Based on these data, one vaccine strategy developed against *Leishmania* infection has been focusing on the use of Th1 responses elicited in immune individuals by defined parasite antigens as indicators of protection. A number of leishmanial proteins have been characterized and evidences from studies in animal models indicate that variable levels of protection can be achieved using defined protein as vaccines [15]–[17]. However, significant differences between mice and humans immune system, in both its innate and adaptive arms exist [18]. Very few candidates have progressed beyond the experimental stage and designing an effective vaccine against leishmaniasis is still a matter of research. One of the candidate vaccine antigens is the PSA which belongs to a unique *Leishmania* family of membrane-bound and secreted proteins. PSA main signature is a specific Leucin Rich Repeats known to be involved in protein-protein interactions and in pathogen recognition [19]. It is an abundant glycolipid-anchored protein on the surface of the promastigote form of most *Leishmania* species [19]–[26]. A water-soluble form released in promastigote culture supernatants of *L. major* [24] and of *L. infantum* [26] has also been described. PSA is involved in parasite attachment and invasion of macrophages [27]. It has been reported to induce Th1-mediated protection against murine leishmaniasis when used as a vaccine [28], [29]. PSA is also specifically recognized by Th1 cells of humans with a history of self-healing CL [30] and by human sera of VL patients [31]. This protein is present in both promastigote and amastigote stages [32], [33] and the presence of PSA family was demonstrated in several *Leishmania* species [19], [21], [34], [35]. PSA proteins are strongly over-expressed in metacyclic promastigotes suggesting an association with the virulence status of the parasites [32]. The known role of PSA proteins in resistance to complement lysis further supports this hypothesis [36]. The interest for the PSA is also supported by our previous [37], [38] and recent studies (unpublished data) showing that sera of dogs vaccinated with *L. infantum* excreted/secreted antigens (ESA) in combination with MDP (muramyl dipeptide), recognized immunodominant antigens belonging to PSA protein family, from *L. infantum* ESA (*Li*PSA-54S; GenBank under Accession number: FJ974055) and from *L. amazonensis* ESA (*La*PSA-38S; GenBank accession number: FJ974054). In the present study, the recombinant native form of *L. amazonensis* PSA protein (*La*PSA-38S), produced in a *L. tarentolae* expression system was evaluated for its ability to induce cellular and humoral immune responses. We took advantage of a multi-disciplinary consortium to assess, using the same tools and samples from individuals with acquired immunity to *L. major*, *L. braziliensis, L. infantum* or *L. donovani*, for PSA immunogenicity and its potential use as a vaccine candidate against the different forms of leishmaniasis.

## Materials and Methods

### 1. Ethics statement

The recruitment and sampling collection of different groups of volunteers, was done in accordance with Good Clinical Practice (GCP), based on the recommendations and approval of the local ethical Committee of each Institute involved in this study (Institut de Recherche pour le Développement (IRD) for France (the recruitment of blood donors and the sample collection were performed at the EFs human blood bank of Bordeaux), Instituto de Salud Carlos III (ISCIII) for Spain (Comité de ética de la investigacion y de bienestar animal), National Institute of Pathology (NIP) for India (Office of ethical committee VMMC & SAFDARJANG hospital New Delhi and Indian council of medical research), Instituto de Medicina Tropical “Alexander von Humboldt” (IMTAvH) for Peru (comité de investigacion y ética de la unidad de capacitacion) and Institut Pasteur de Tunis (IPT) for Tunisia (Comité d'éthique de l'Institut Pasteur de Tunis). A written informed consent was obtained from all subjects involved in this study.

### 2. Study population and samples

Well-characterized endemic foci, blood banks and medical structures (hospitals, medical departments and public health centers) were identified in each country (India, Peru, Spain, France, and Tunisia). Human groups (cured individuals and healthy individuals with no history of leishmaniasis but with a probable asymptomatic infection and therefore considered as immune) were recruited from endemic areas for VL and CL, based on the following defined inclusion criteria (i) living in endemic foci to *L. major, L. infantum, L. braziliensis* or *L. donovani* (ii) well documented medical records for cured groups (iii) presence of typical scars for cured CL group and (iv) high IFN-γ response to Soluble *Leishmania* Antigens (SLA) (>300 pg/ml) for healthy individuals with a probable asymptomatic infection. Patients with active CL or VL were identified in health structures in charge of patient management and had specific clinical symptoms. Healthy individuals recruited in low or non-endemic areas, or in blood banks with no history of leishmaniasis and no or low IFN-γ response to SLA (<100 pg/ml) were considered as non immune/naives. Exclusion criteria were immunosuppressive diseases other than leishmaniasis, long term treatment and pregnancy. The different human groups used in this study are detailed in table 1. Heparinized blood was collected from a total of 104 donors and 82 controls (Table 1).

**Table 1:** Study population.

Country	Human groups*	Number of individuals	Parasite species
India	CVLd	16	*L. donovani*
	aVLd	25	*L. donovani*
	HLR-I	19	
Peru	CCLb	8	*L. braziliensis*
	aCLb	21	*L. braziliensis*
	HLR-P	10	
Tunisia	CCLm	25	*L. major*
	HHR-Lm	20	*L. major*
	HHR-LiT	18	*L. infantum*
	aVLiT	12	*L. infantum*
	HLR-T	16	
France	HHR-LiF	8	*L. infantum*
	HLR-F	28	
Spain	HHR-LiS	9	*L. infantum*
	aVLiS	4	*L. infantum*
	HLR-S	9	

The recruitment and sampling collection (186 donors) of different groups (patients, cured, immunes without clinical symptoms and naives) were performed in endemic and non-endemic areas in each country.

*CVLd: Cured Visceral Leishmaniasis due to *L. donovani* (India), aVLd: active Visceral Leishmaniasis due to *L. donovani* (India), HLR-I: Healthy Low Responders from India, CCLb: Cured Cutaneous Leishmaniasis due to *L. brasiliensis* (Peru), aCLb: active Cutaneous Leishmaniasis due to *L. brasiliensis* (Peru), HLR-P: Healthy Low Responders from Peru, CCLm: Cured Cutaneous Leishmaniasis due to *L. major* (Tunisia), HHR-Lm: Healthy High Responders living in an endemic area for *L. major* (Tunisia), HHR-LiT: Healthy High Responders living in an endemic area for *L. infantum* (Tunisia), aVLiT: active Visceral Leishmaniasis due to *L. infantum* (Tunisia), H-T: Healthy Low Responders from Tunisia, HHR-LiF: Healthy High Responders living in an endemic area for *L. infantum* (France), HLR-F: Healthy Low Responders from France, HHR-LiS: Healthy High Responders living in an endemic area for *L. infantum* (Spain), aVLiS: active Visceral Leishmaniasis due to *L. infantum* (Spain), HLR-S: Healthy Low Responders from Spain.

### 3. Production and purification of *La*PSA-38S recombinant protein

Genomic DNA of *L. amazonensis* (MHOM/BR/76/LTB-012/clone 1), cultivated in RPMI 1640 medium (Gibco-BRL, UK) and supplemented with 10% heat-inactivated FBS (Gibco-BRL, UK), 10 mM HEPES, 100 U/ml penicillin, and 100 µg/ml streptomycin (Gibco-BRL, UK) at 26°C, was extracted by phenol: chloroform: isoamyl alcohol extraction (1∶1∶1) and ethanol precipitation. The *La*PSA-38S gene (GenBank accession number: FJ974054) was amplified with forward primer, F-PSA-38S (5′-CCATGGCGCAGTGCGTGCGTCGG-3′) and reverse primer, R-PSA-38S (5′-GCGGCCGCGTGATGGTGATGGTGATGATCGTGGTTCGCCAG-3′), containing *Nco*I and *Not*I restriction sites in each 5′ end (underlined) by PCR reaction. The purified PCR product was cloned in pCR2.1-TOPO TA vector using TOPO TA cloning Kit (Invitrogen) following the manufacturer's procedures. The transformed cells *E. coli* TOP10 (Invitrogen) were screened for the presence of recombinant plasmid with the *La*PSA-38S insert by gene-specific PCR and analysis with *Nco*Iand *Not*Irestriction enzymes and isolated positive clones were sequenced. The insert was removed by *Nco*Iand *Not*I digestion and subcloned into the *Nco*I and *Not*I insertion site of *Leishmania* expression vector pF4X1.4sat1 allowing selection with the antibiotic Nourseothricin (Jena Bioscience, Germany) to create the recombinant pF4X1.4-*La*PSA-38S plasmid. The resulting construct, encoding a full sequence of *La*PSA-38S secreted protein fused to a C-terminus His_6_ tag. *L. tarentolae* promastigotes were successfully maintained in continuous culture by successive passages of 5×10^5^ flagellates/ml every week into 10 ml of completely chemically defined medium (CDM/LP) free of serum, macromolecules, proteins and cell contaminants as previously described [39], [40]. For stable integration of the expression cassette into the 18S ribosomal RNA (ssu) locus, 10 µg of pF4X1.4sat1 plasmid containing *La*PSA-38S gene was digested by *Swa*I restriction enzyme. Transfections of *L. tarentolae* promastigotes were performed by electroporation in 2 mm cuvettes using a Gene Pulser II (Biorad) and a single pulse (5–6 msec) with the settings 450 V and 450 µF. After pulse, cells were transferred into a fresh CDM/LP medium. 100 µg/ml of Nourseothricin was added 24 h after electroporation to select stable transformants. After a week of culture only nourseothricin-resistant cells survived. Transgenic cells were selected as single colonies on the supplemented CDM/LP-agar medium containing 100 µg/ml Nourseothricin. To confirm the integration of the *La*PSA-38S containing cassette into the *Leishmania* genome, PCR was performed on the genomic DNA of wild type and transgenic cells with *ssu* forward primer F3001 and *aprt* reverse primer A1715 (Jena bioscience, Germany). Mass culture and protein purification were manufactured by Virbac in GMP conditions. Culture amplifications were performed on CDM/LP medium. The culture supernatant containing *L. amazonensis* excreted secreted *La*PSA-38S, released by the parasite during its growth, was recovered at the late stationary phase of growth. The only proteins contained in the medium were of parasitic origin and were found with their native conformation because they were naturally excreted-secreted by *Leishmania* parasites. Excreted secreted PSA was purified from concentrated culture supernatant by Ni-NTA affinity chromatography.

### 4. Preparation of total soluble Leishmania promastigote antigens (TSLA)

All antigen extracts were prepared from promastigote stationary phase parasite cultures of *L. donovani*, *L. braziliensis*, *L. major* and *L. infantum*. Each consortium partner used local TSLA (*L. major* TSLA in Tunisia, *L. infantum* TSLA in France and Spain, *L. braziliensis* TSLA in Peru and *L. donovani* TSLA in India) and TSLA from *L. donovani* (TSLA Ldd8) (MHOM/IN/80/Ldd8Cl_2_ *L. donovani* strain (Ldd8)), as a standard preparation. TSLA were obtained from washed parasites in 1x phosphate-buffered saline (PBS), centrifuged at 1000×g/10 min at 4°C and supernatants were removed. The pellets were resuspended in lysis buffer (50 mM Tris/5 mM EDTA/HCl, pH7. 1 mL/1×10^9^ parasites), subjected to three rapid freeze/thaw cycles followed and to three pulses of 20 seconds/40W with sonicator. Samples were centrifuged at 5000×g for 20 min at 4°C, and supernatants were collected, aliquoted and stored at −80°C until use. Protein quantification was performed using Bradford method.

### 5. Cell culture and stimulation

Peripheral blood mononuclear cells (PBMC) were isolated from blood by density centrifugation through Ficoll-Hypaque (GE Healthcare Bio-Sciences AB, Uppsala, Sweden).

The cells were cultured in RPMI 1640 supplemented with 10% heat inactivated FBS, 100 IU/mL penicillin, 100 µg/mL streptomycin, 2 mM L-glutamin, 50 µM 2-mercaptoethanol, 1 mM sodium pyruvate and 1X amino acid non essential. Briefly, cells were plated in 96 well (TPP, Switzerland) and were kept with media alone (unstimulated) or stimulated with 10 µg/mL of Phytohemagglutinin (PHA) (Sigma-Aldrich) as a positive control or with TSLA (10 µg/mL) or with *La*PSA-38S (10 µg/mL), in a 5%CO_2_ humidified atmosphere at 37°C for 5 days.

### 6. Cytometric Bead Array assay (CBA)

IFN-γ, granzyme B, tumor necrosis factor-alpha (TNF-α) and Interleukin-10 (IL-10), were detected and quantified from culture supernatants (50 µl) of PBMC exposed for 120 h to the following stimuli: PHA-M (10 µg/ml), TSLA (10 µg/ml) or *La*PSA-38S (10 µg/ml), using the BD CBA Human Soluble Protein Flex Set system and according to the instructions of the manufacturer (BD Biosciences). It should be noted that granzyme B was not analyzed in individuals immune to *L. braziliensis* infection. In order to quantitate samples, the BD CBA Human Soluble Protein Flex Standard was performed for each cytokine and in each experiment. Data were acquired by flow cytometry (FACSCalibur or FACSCanto) using 2-color detection. Flow Cytometric Analysis Program Array (FCAP Array; BD Biosciences) software was required for analyzing samples [41].

### 7. Cell phenotyping

Freshly isolated PBMC were stimulated with PMA (50 ng/ml)/ionomicyn (10^−6^ M) or PHA at 10 µg/ml for 6 h (positive control) or with TSLA at 10 µg/ml or with *La*PSA-38S at 10 µg/ml for 120 h and, or kept with medium alone. Cells were treated with Golgistop (BD Biosciences) for the last 6 hours of culture, then washed and incubated with antibodies: FITC CD3, PerCPcy5.5 CD4, APC-H7 CD8 or PerCPcy5.5 CD8, and PE-Cy7 CD69 or PE CD69 (BD Biosciences), for 20 minutes at 4°C. For intracellular IFN-γ detection, cells were fixed and permeabilized using BD Cytoperm/cytofix plus kit (BD Biosciences) according to manufacturer's instructions and labeled with PE-anti-IFN-γ mAb (intracellular formulation) (BD Biosciences). Analysis was performed with FACSCalibur using CellQuest Pro software or FACS canto II flow cytometer using DIVA software. FITC mouse IgG1, PerCPCy5.5 mouse IgG1, mouse IgG1PE, PE mouse IgG2a (intracellular formulation) (Biosciences) were used as isotype controls for acquisition by FACSCalibur. For acquisition by FACSCanto, BD CompBeads Set Anti-Mouse Ig, κ (Anti-Mouse Ig, κ/Negative Control (FBS) Compensation Particles Set) (BD Biosciences) were used.

### 8. Detection of antibodies specific for *La*PSA-38S

An enzyme-linked immunosorbent assay (ELISA) test was performed to detect specific antibodies to TSLA Ldd8 and *La*PSA-38S in individual serum samples from the different studied groups (Table 2). In order to improve antibodies detection, ELISA protocol was optimized by combining antigens concentrations and anti-serum dilutions. Briefly, 96 wells plates (NuncMaxisorp Immuno Plates, PolyLabo, Strasbourg, France) were coated with 100 µl/well of 2 or 5 µg/ml *La*PSA-38S and TSLA overnight at 4°C. Plates were washed three times with PBS, 0.1% Tween 20 (PBS-T), pH 7.4 and blocked with 200 µl/well of PBS containing 0.1% Tween 20 and 3% BSA for 1 h at 37°C. After washing with PBS-T, diluted sera (1/100, 1/150 and 1/200 in PBS-T) were added (100 µl/well) and incubated for 1h30 at 37°C. Plates were then washed with PBS-T and 100 µl/well of diluted HRP-conjugated anti-human IgG in washing buffer were added for 30 min at 37°C (1/5000). Plates were developed with the 100 µl/well of Sigma Fast *o*-phenylene diamine dihydrochloride (OPD) for 15 min. The reaction was stopped with 50 µl/well of 2 N HCl. The absorbance was measured at 492 nm. All sera were tested in duplicate/triplicate, and the mean value was recorded.

**Table 2:** Frequency of CD4+ and CD8+ T cells after SLA and LaPSA-38S stimulation.

	*Medium*				*PMA/iono or PHA*				*SLA*				*LaPSA-38S*			
Groups	CCLm	HHR-Lm	HHR-LiS	HLR-S	CCLm	HHR-Lm	HHR-LiS	HLR-S	CCLm	HHR-Lm	HHR-LiS	HLR-S	CCLm	HHR-Lm	HHR-LiS	HLR-S
**CD4+T cells**	64,4±5,97	56,75±6,01	51,2±5,01	44,8±10,22	53,9±9,45	43,4±12,98	48,9±6,72	39,6±7,93	57,7±10,7	57,2±4,70	52,1±9,50	43,5±11,30	57,4±10,41	59,5±3,48	52,7±5,49	43,2±9,08
**CD8+T cells**	25,15±6,88	27±6,00	20,6±6,59	22,9±5,91	24,4±5,66	39,6±20,82	41,6±10,93	47,8±9,83	21,3±6,80	25,4±3,10	20,7±5,50	21,3±6,10	24,5±4,31	25,2±4,95	20,6±5,03	23,5±6,25

Phenotypic analysis of *La*PSA-38S and SLA-specific CD4+ and CD8+ was performed for PBMC among CD3+ population, in groups in which we observed a significant and specific *La*PSA-38S-induced IFN-γ response (CCLm: n = 7, HHR-Lm: n = 6, HHR-LiS: n = 7). Naive individuals (HLR-S: n = 14) were also analyzed. Data are means percentage ± SD.

### 9. Statistical analysis

Data from all partners were collected into a unique database using the open source software Epidata 3.1 (Epidata Association, Denmark http://www.epidata.dk/) and checked for consistency. Individuals with missing data or exclusion criteria were deleted. Data analysis was then performed with Stata statistical software (StataCorp. 2009. Stata Statistical Software: Release 11. College Station, TX: StataCorp LP.). We used non-parametric statistical tests because of the low number of individuals in some groups and of the heterogeneity of the standard deviation between groups. Results are expressed as mean±standard deviation (SD) and a p-value of <0.05 was considered significant in all cases. In the statistical analysis of cytokine level and phenotyping, we used Wilcoxon signed-rank test to compare median levels of cytokines or percentage of cells producing IFN-γ after different stimulations in paired data. Kruskal-Wallis rank test was used for inter-groups analysis on normalized data after deducting the non-stimulated value. Correlations were performed using pairwise correlations of variance test. For humoral response analysis, we used Kruskal-Wallis rank test to compare the median production of total IgG between different stimulations. We estimated a threshold of positivity for each country by calculating a cut-off using the following formula:
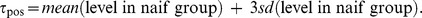



Individuals with a level of IgG after stimulation higher than τ_pos_ were considered as positive responders. For the statistical analysis of sub-classes, results were compared only for positive individuals

## Results

### 1. Production of *La*PSA-38S in *L. tarentolae*

**Figure 1:**
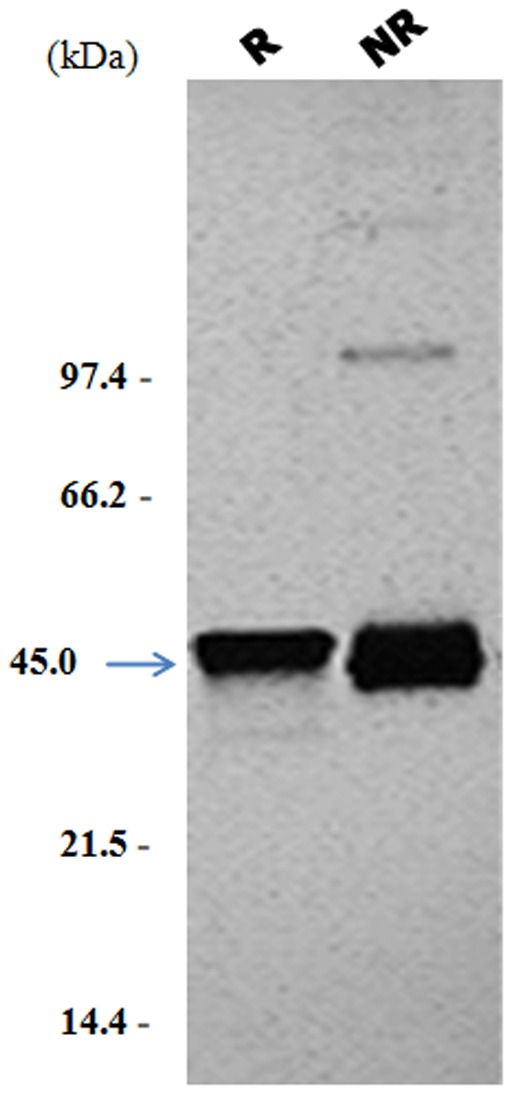
SDS-PAGE and silver staining of purified recombinant *La*-PSA38S protein expressed in *L. tarentolae*. Purified *La*-PSA38S was separated by electrophoresis in a 12% SDS-PAGE and silver stained under reducing (R) and non reducing (NR) conditions. The arrow indicates the position of *La*-PSA-38S (45 kDa) and Molecular Weight markers were marked in kDa.

In this study, we generated a recombinant *L. tarentolae* strain expressing exogenously the *L. amazonensis La*PSA-38S gene. The resulting construct encoded a full sequence of *La*PSA-38S secreted protein fused to a C-terminus His_6_ tag. *La*PSA-38S protein purity was assessed by SDS–PAGE using 12% polyacrylamide Ready Gels (Bio-Rad) under reducing and non reducing conditions and after silver staining. Immobilized Ni^2+^ affinity chromatography of culture supernatant containing excreted/secreted extracts from recombinant *L. tarentolae*, yielded a preparation featuring a purified recombinant *La*PSA-38S protein migrating as a 45 kDa species in SDS–PAGE (Figure 1). The extra band observed in figure 1 was identified as a dimer form of *La*PSA-38S as suggested by a proteomic study of purified *La*PSA-38S using a combination of 1D gel electrophoresis and LC-MS/MS (coupling between liquid chromatography and tandem mass spectrometry) (Bras-Gonçalves et al., manuscript in preparation).

### 2. IFN-γ, granzyme B, TNF-α, and IL-10 levels in response to *La*PSA-38S protein in immune individuals

**Figure 2:**
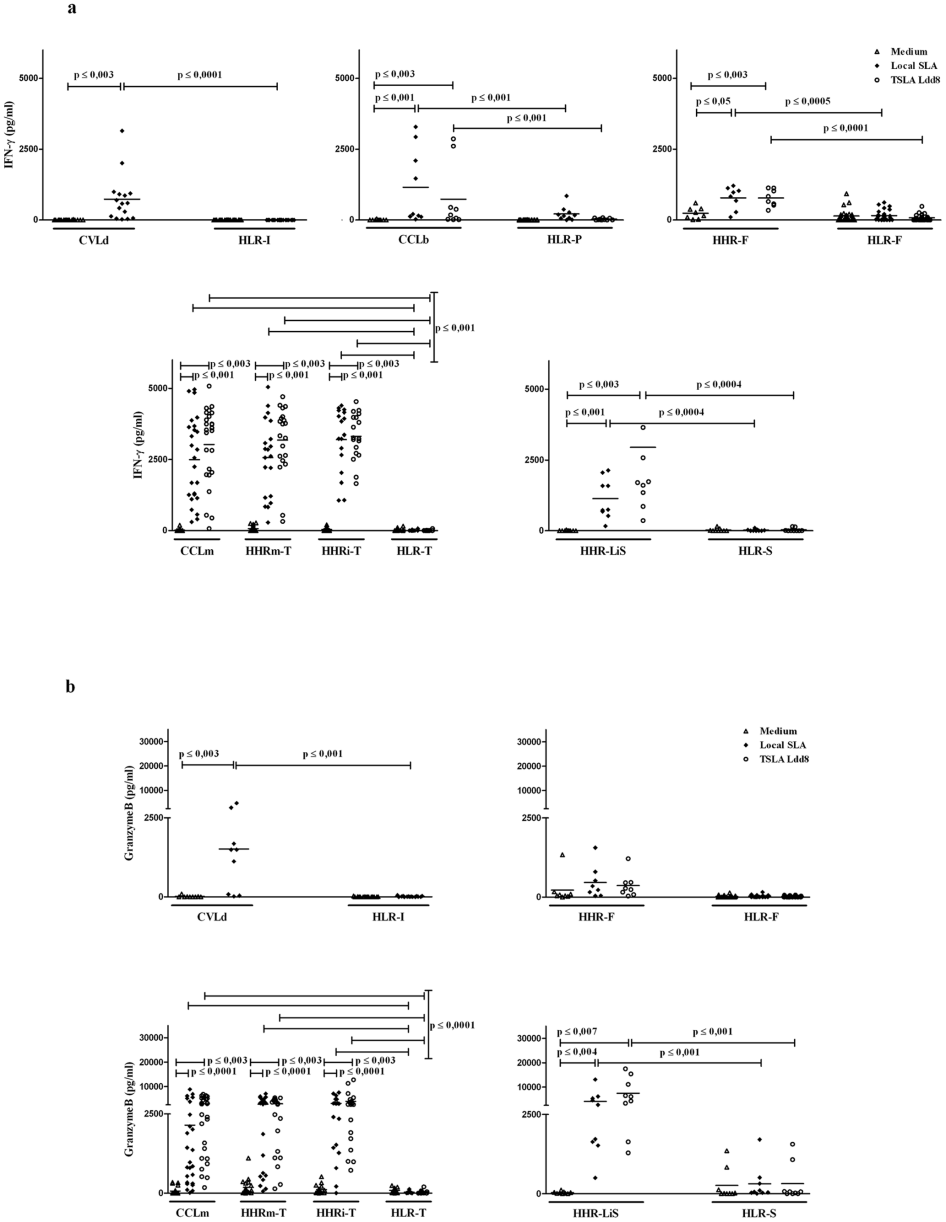
Total Soluble *Leishmania* antigen (TSLA) specific IFN-γ, granzyme B, TNF-α responses. IFN-γ (Fig. 2a), granzyme B (Fig. 2b), were detected and quantified from culture supernatants of PBMC exposed for 120h to local TSLA (10 µg/ml) and TSLA Ldd8 (10 µg/ml), by Cytokine Beads Array test (CBA) using Flow cytometry. Statistically significant differences between stimulated and non stimulated cultures and between groups (p≤0.03) are showed.

**Figure 3:**
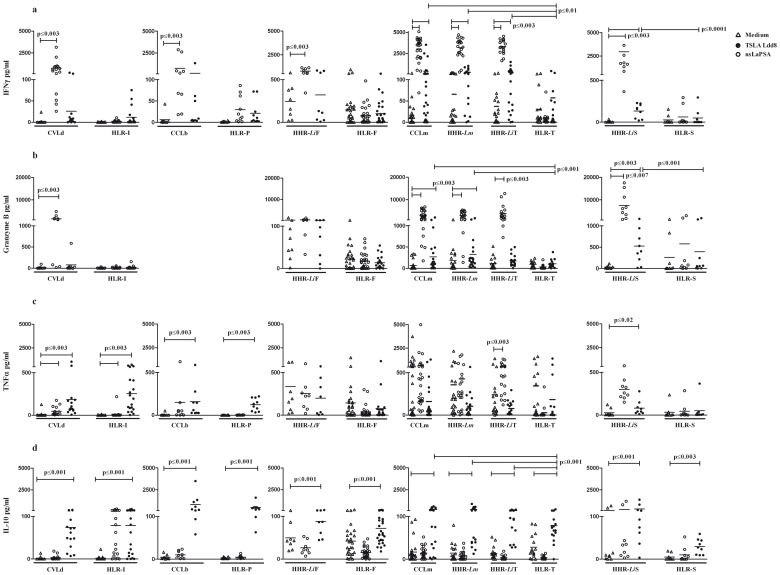
*La*PSA-38S specific IFN-γ, granzyme B, TNF-α and IL-10 responses. IFN-γ (Fig. 3a), granzyme B (Fig. 3b), TNF-α (Fig. 3c) and IL-10 (Fig. 3d) were detected and quantified from culture supernatants of PBMC exposed for 120 h to SLA (10 µg/ml) or *La*PSA-38S (10 µg/ml) using Cytokine Beads Array test (CBA). Data were analyzed by Flow cytometry. PHA (10 µg/ml) was used for all cultures as positive control (data not shown). Statistically significant differences between stimulated and non stimulated cultures (p≤0.003) and between groups (p≤0.01) are showed.

The supernatants of TSLA or *La*PSA-38S-stimulated PBMC were assayed for IFN-γ, granzyme B, TNF-α and IL-10 production (Figure 2, Figure 3). PBMC were isolated from (i) individuals cured from *L. major* (CCLm), *L. braziliensis* (CCLb) or *L. donovani* (CVLd) infection, (ii) healthy individuals living in endemic areas and showing high IFN-γ response to TSLA, considered as immune (HHR-*Lm*: Healthy High Responder living in endemic area to *L. major* infection and HHR-*Li*, HHR living in endemic area to *L. infantum* infection) and (iii) healthy individuals living in low or non-endemic areas and showing low or no IFN-γ response to TSLA, considered as non immune/naive (HLR-T: Healthy Low Responders from Tunis; HLR-F from France; HLR-S from Spain; HLR-I from India and HLR-P from Peru). HHR-*Li* individuals were recruited from three different areas (France: HHR-*Li*F; Spain: HHR-*Li*S; Tunisia: HHR-*Li*T). *La*PSA-38S induced significant (when compared to non stimulated cultures, p≤0.003) and specific (when compared to corresponding naive groups, p≤0.01) levels of IFN-γ in PBMC cultures of CCLm, HHR-*Lm*, HHR-*Li*T and HHR-*Li*S (Mean ± SD: 410.9±913.1 pg/ml; 297.2±306.9 pg/ml; 297.2±423.2 pg/ml; and 127.3±79.1 pg/ml, respectively) (Figure 3a). No significant IFN-γ levels were detected in HHR-*Li*F or in naive groups HLR-T, HLR-F or HLR-S. Low but significant IFN-γ levels were observed in CCLb and CVLd but also in their related naive groups (HLR-P and HLR-I, respectively) (Figure 3a). *La*PSA-38S also induced significant and specific (p≤0.04) granzyme B levels in CCLm (235.7±448.5), HHR-*Lm* (311.7±345.9) and HHR-*Li*S (492.8±451) (Figure 3b). Granzyme B was not observed in naive individuals. The levels of IFN-γ and granzyme B were significantly higher in the supernatants of all immune groups (CCLm: 3027.1±1356.4 and 2852.2±2035.9; CCLb: 740.2±1145.6, granzyme B was not tested; CVLd: 740.1±824.4 and 1511.1±1549.5; HHR-*Lm*: 3182.3±1198.4 and 2608.5±1818.1; HHR-*Li*T: 3311.8±818.3 and 3873.6±3401.8; HHR-*Li*F: 778.8±299.3 and 364.5±377.9; HHR-*Li*S: 2946.3±3773.7 and 7478±5902.6; respectively) compared to naives (p≤0.007) after TSLA Ldd8 stimulation (Figure 2a and b). It should be noted that no significant differences were observed in IFN-γ and granzyme B responses between local TSLA and TSLA Ldd8 (Figure 2a and b). Significant TNF-α levels (p≤0.02) were observed in CCLb (158.5±216.2), CVLd (183.9±271.4) and HHR-*Li*S (54.7±46), but also in some naive groups (HLR-P: 122.2±72 and HLR-I: 253.7±227.3) after *La*PSA-38S stimulation (Figure 3c). TSLA Ldd8 induced significant and specific TNF-α production (p≤0.03) in the same groups but also in HHR-*Li*T (CCLb: 149.3±333.5; CVLd: 47.2±50.8; HHR-*Li*S: 275.2±152.8 and HHR-*Li*T: 529.2±359.9) (Figure 3c). PBMC from all immune and naive groups showed significant levels of IL-10 (p≤0.02) in response to *La*PSA-38S (immune groups: CCLm: 218.5±184.3; CCLb: 797.8±1110; CVLd: 74.1±54.9; HHR-*Lm*: 233.4±241.7; HHR-*Li*T: 104.5±56.2; HHR-*Li*F: 87.9±37.9; HHR-*Li*S: 175.7±244.8; naive groups: HLR-T: 73.5±54.2; HLR-P: 449.4±432.6; HLR-I: 78.8±83.6; HLR-F: 71.8±38.3; HLR-S: 24.5±17.6) (Figure 3d). IL-10 levels were significantly higher in CCLm, HHR-*Lm* and HHR-*Li*T compared to corresponding naive groups (HLR-T) (p≤0.009). No IL-10 was observed in response to TSLA. No difference was observed in the cytokine levels between immune groups, except for *La*PSA-38S-induced granzyme B levels which were significantly higher in HHR-*Li*S compared to CCLm and HHR-*Lm* (p = 0.02 and 0.04, respectively).

Interestingly, strong positive correlations were found between IFN-γ and granzyme B production in CCLm group (r = 0.82; p = 0.0000) and between granzyme B and IL-10 production in HHR-*Li*S group (r = 0.7; p = 0.03), in response to *La*PSA-38S. IFN-γ and granzyme B productions were also highly correlated in response to TSLA Ldd8 in CCLm and HHR-*Li* groups (r = 0.67; p = 0.0002 and r = 0.76; p = 0.01; respectively).

### 3. Phenotyping results

**Figure 4:**
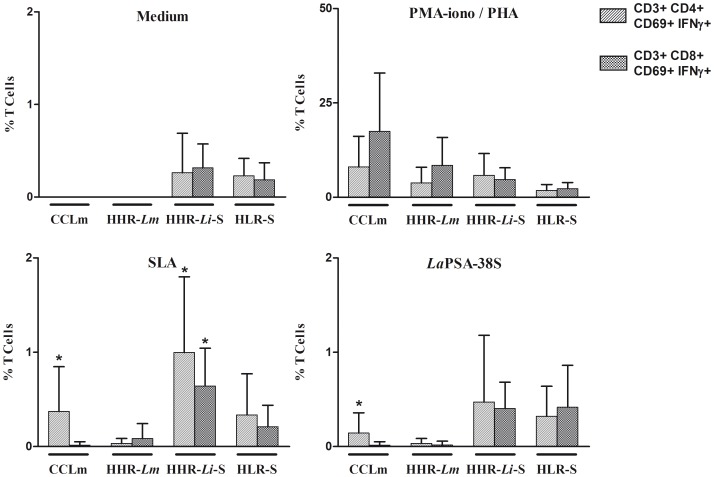
Phenotype of IFN-γ producing cells. PBMC were stimulated with PMA (50 ng/ml)/ionomicyn (10^−6^ M) or PHA (10 µg/ml) for 6 h (positive controls), TSLA Ldd8 (10 µg/ml) or *La*PSA-38S (10 µg/ml) for 120 h. For intracellular IFN-γ detection, cells were treated with Golgistop for the last 6 hours of culture, fixed and permeabilized using BD Cytoperm/cytofix kit, and analyzed by flow cytometry. Results represent the frequency of IFN-γ producing cells within the CD3^+^CD4^+^CD69^+^ and CD3^+^CD8^+^CD69^+^ cell populations. * Statistically significant differences from stimulated and non stimulated cultures (p<0,05).

Phenotypic analysis of *La*PSA-38S and TSLA Ldd8-specific CD4+ and CD8+ cells producing IFN-γ was performed in groups in which we observed a significant *La*PSA-38S-induced IFN-γ response (CCLm: n = 7, HHR-*Lm*: n = 6, HHR-*Li*S: n = 7) and in naive individuals (HLR-S: n = 14) (Figure 4).

We showed a significantly higher percentage of CD4+ T cells producing IFN-γ (mean +/− SD: 0.14 +/−0.21%) among activated CD4+ T cells (57.4+/−13.9%), after *La*PSA-38S stimulation, only in CCLm individuals, in comparison to non stimulated cultures (p = 0.049), (Figure 4, table 2). A strong positive correlation was found between the percentage of IFN-γ-producing CD4+ T cells and IFN-γ production in cell culture supernatants assessed by CBA analysis, in response to *La*PSA-38S antigen (r = 0.97, p = 0.0003). No significant increase was observed in the percentage of IFN-γ-producing CD8+ T cells in tested immune groups, in response to *La*PSA-38S, compared to non stimulated cultures (Figure 4). A significant increase was showed in the percentage of IFN-γ-producing CD4+ T cells in CCLm (0.37+/−0.47%) and HHR-*Li*S (0.87+/−0.74) and of IFN-γ-producing CD8+ T cells in HHR-*Li*S (0.53+/−0.4), compared to non stimulated cultures (p = 0.02), in response to TSLA Ldd8 stimulation (Figure 4).

### 4. Humoral response to *La*PSA-38S

Anti-*La*PSA-38S total IgG responses were analyzed by using ELISA tests in sera from (i) patients with active disease (aVLd, aVLiT, aVLiS, aCLb), (ii) cured individuals (CVLd, CCLm, CCLb), (iii) healthy individuals with high IFN-γ response to TSLA Ldd8 as immune with no clinical symptoms (HHR-*Lm*, HHR-*Li*T, HHR-*Li*F, HHR-*Li*S) and healthy individuals with low IFN-γ response to TSLA Ldd8 as naïve controls (Table 3). The cut-off value of reactivity for *La*PSA-38S antigen was calculated as the mean plus 3 SD of the OD values observed in naive controls from each country. These cut-off values allowed us to distinguish between positive and negative sera and consequently to estimate performance parameters of each ELISA test (Table 3). Globally, the results indicated that *La*PSA-38S antigen does not constitute a target for the humoral response in the different studied groups. The percentage of positivity was very low ranging from 0% to 12.5%. However, an exception was observed with the aVLiS group where *La*PSA-38S was recognized by 50% of tested sera. In contrast and as expected, highest positivity was observed using TSLA antigen. Best results were obtained with sera from individuals with active VL diseases which 100% react with TSLA Ldd8.

**Table 3:** Total IgG prevalence against *La*PSA-38S in different endemic areas of leishmaniasis.

Endemic Area	Ig	Group of individuals	Tested sera	Percentage of positive sera (%)	
				SLA	*La*PSA
**India**	Total IgG	CVLd	13	92,3	**-**
		**aVLd**	25	100	**8**
		HLR-I	9	-	**-**
**Peru**	Total IgG	CCLb	9	-	**-**
		aCLb	21	4,8	**-**
		HLR-P	10	-	**-**
**Tunisia**	Total IgG	**CCLm**	19	68,4	**5,3**
		**HHR-Lm**	20	15	**5**
		**HHR-Li**	15	40	**6,7**
		aVLiT	12	100	**-**
		**HLR-T**	17	5,9	**5,9**
**France**	IgG	**HHR-Li F**	8	12,5	**12,5**
		**HLR-F**	28	-	**3,5**
**Spain**	IgG	HHR-Li S	9	-	**-**
		**HLR-S**	9	-	**11,11**
		**aVLiS**	4	100	**25**

The cut-off value of reactivity for *La*PSA-38S antigen was calculated as the mean plus 3 SD of the OD values observed in naive controls from each country. These cut-off values allowed us to distinguish between positive and negative sera and consequently to estimate performance parameters of each ELISA test.

## Discussion

Few vaccine candidates have progressed beyond the experimental stage in animal models of leishmaniasis (mice, hamsters). This is consistent with the difficulty in developing a suitable animal model that reproduces the features of the human disease. Direct extrapolation of data from animal models to human disease is often controversial and the protective immune responses controlling experimental infection may not reflect those required to prevent leishmaniasis in endemic areas. The purpose of this study was to evaluate the ability of a PSA antigen to elicit an *ex vivo* cellular immune response in PBMC of non exposed and previously exposed human subjects with and without evidence of protective immunity. The rationale is that antigens that elicit immune responses correlating with protection or cure (e.g. significant IFN-γ production in previously exposed and cured individuals) may be good candidates for prophylactic and/or therapeutic vaccines.

We have previously demonstrated that naturally ESA purified from culture supernatants of *L. infantum* promastigotes formulated with an adjuvant were able to induce a long lasting Th1-mediated protection against experimental and natural canine VL [19], [32], [37], [38]. Interestingly, a native soluble *L. infantum* PSA has been identified as the active constituent of ESA that reproduce the observed protective immunity when used as a vaccine in dogs (unpublished data). PSA is present in both promastigote and amastigote stages and is over-expressed in stationary phase parasites [19], [32], [33]. It is highly conserved in its N-terminal and C-terminal parts, among *Leishmania* species, which is a requisite for ensuring cross-species protection (unpublished data). PSA isolated from *L. amazonensis* promastigotes as well as recombinant vaccinia viruses expressing this protein have been demonstrated to elicit a protective immune response against infection with *L. amazonensis* in BALB/c mice [28], [42]. Moreover, PSA-2 polypeptides purified from L. major and plasmid DNA encoding PSA-2, but not recombinant PSA-2 purified from *E. coli*, provided significant protection against *L. major* infection in BALB/c mice [29], [43]. Our aim was to identify a human vaccine candidate targeting *Leishmania* species that cause CL and VL. We used a recombinant native *L. amazonensis* PSA (*La*PSA-38S), as a potential candidate vaccine. Despite the fact that the amino sequence alignment of *La*PSA-38S with PSA proteins from several *Leishmania* species, shows that these proteins are not 100% identical, the N-terminal and C-terminal regions present highly conserved segments. However, the number of Leucine Rich Repeat motifs located in the central region was variable [19]. Nevertheless, these proteins belong to the same unique PSA family according to various well known criteria which constitute the PSA family unique signature [19]. Thus, all PSA might be considered as highly similar regarding their protein primary structure since they correspond to the same architecture depicted by a precise domain organization which is well conserved among PSA from different *Leishmania* species [19].

*La*PSA-38S was produced in a non-pathogenic *L. tarentolae* recombinant expression system. This expression system allows the conservation of *Leishmania-*type post-translational modifications including glycosilation, phosphorylation and disulfide bond formation. Moreover, the *L. tarentolae* expression system, in addition to being non-pathogenic to mammals, is an ideal expression system to produce native proteins of *Leishmania* [44]. A multi-disciplinary consortium has been set-up including groups from Europe (France and Spain for *L. infantum* infection) and from different endemic countries where both VL and CL are highly prevalent (India for *L. donovani*, Peru for *L. braziliensis* and Tunisia for *L. major* and *L. infantum* infections). We demonstrated that *La*PSA-38S induced similar significant and specific levels of IFN-γ in individuals immune to *L. major* and *L. infantum* infection. Healthy high IFN-γ responders had equivalent responses to the cured group. A non specific IFN-γ response to *La*PSA-38S stimulation was observed in *L. braziliensis* or *L. donovani* immune individuals. Analysis of the phenotypes of *La*PSA-38S-stimulated T cells showed a significant increase in the percentage of IFN-γ producing CD4+ T cells detected only in cured individuals. Furthermore, a strong positive correlation was found between the percentage of IFN-γ-producing CD4+ T cells and the IFN-γ production determined by CBA. Data on the PSA immunogenicity in humans are scarce. One study has shown that PSA from *L. amazonensis* was able to stimulate T cell proliferation in leishmaniasis patients [45]. To our knowledge, only one study reported that PSA from *L. major* was able to induce strong proliferative responses and high IFN-γ and TNF-α levels in individuals with a past history of self-healing CL [30]. Healing from human leishmaniasis is generally associated with an appropriate Th1 response characterized by IFN-γ production. Thus, our finding showing that *La*PSA-38S stimulates high levels of IFN-γ in individuals with immunity to *L. major* or *L. infantum* infection, suggests that this protein may be associated with protective immunity. With regard to IL-10 production, we showed that, in addition to its Th1 response-inducing activity, *La*PSA-38S induces significant IL-10 levels in individuals with immunity to *L. major* or *L. infantum* infection, and to a lesser extent in naïve groups. IL-10 is an anti-inflammatory cytokine with broad immune-regulatory functions, produced by different cell populations including dendritic cells, macrophages, Th2 and different regulatory CD4+ T subsets [46]. The regulatory effects of IL-10 on immune responses and pathology have been frequently reported [47]. Heterogeneity of CD4+ T cell cytokine response has complicated the definition of the immune correlates of vaccine-mediated protection [48]. Recently, the balance between pro-inflammatory IFN-γ/TNF-α and regulatory IL-10 cytokines has been shown to be involved in the outcome of human leishmaniasis and in the prediction of vaccine success [17]. Therefore, the capacity of *La*PSA-38S to induce IL-10 does not exclude this protein as a potential candidate vaccine. We also detected significant and specific levels of granzyme B in *L. major* and *L. infantum* immune individuals. Very few reports have investigated the involvement of granzyme B in human leishmaniasis. While an increase of granzyme B activity have been associated with a good prognosis in human CL caused by *L. major* or *L. mexicana*, [49], [50], CD8+ granzyme B+ T cells have been reported to mediate tissue injury in CL caused by *L. braziliensis* [51]. Granzyme B is a serine protease expressed in human T CD8+ and T CD4+ cells as well as in Treg cells [52]. It has been first implicated in apoptosis of target cells [53]. Recently, several studies have highlighted a new inflammatory role of granzyme B through the regulation of cytokine expression and processing and regulatory T cells activity [53]-[56]. To our knowledge, this is the first description of a *Leishmania* antigen-induced granzyme B response in humans immune to *Leishmania* infection. The increase of granzyme B, in response to a heterologous prime boost vaccination of mice using the LACK p36 antigen suggests its contribution to the disease control [57]. Our data also show strong positive correlations between granzyme B and IFN-γ levels in response to *La*PSA-38S, in individuals with cured *L. major* CL, which could be associated with protective immunity.

We also showed in our study that *La*PSA-38S induced a mixed Th1 and Th2/Treg cytokine profile in individuals with immunity to *L. major* and *L. infantum* infection but not in individuals with immunity to *L. braziliensis* or *L. donovani*. Data on conserved *Leishmania* antigens enabling cross-species protection were reported in murine models [58]–[60]. However, absence of cross-species protection was more frequently described [61]–[63]. The absence of cross-protection between *Leishmania* species could be explained by the biological differences between *Leishmania species* and by the level of sequence homology of the *Leishmania* proteins used. Indeed, while the *L. major* and *L. infantum* proteins-coding sequences are conserved, the new world *L. braziliensis* proteins-coding sequences are more divergent [64]. Finally, humoral responses analysis results clearly show that *La*PSA-38S antigen did not induce a significant antibody response in individuals that display different clinical manifestations and forms of leishmaniasis. These results are in accordance with other studies describing reactivities of human VL sera to PSA antigen [32]. However, in our case, we cannot exclude that this low recognition may be due to the amino-acids sequence divergence between *La*PSA-38S (used in our experiment) and PSA from *L. major*, *L. infantum* and *L. donovani*. In conclusion, we have demonstrated here that, in spite of differences in disease forms induced by *L. major* and *L. infantum*, the *La*PSA-38S protein was able to induce a similar Th1 and Th2/Treg profile in individuals immune to infection by these two species. To our knowledge, this is the first description of PSA-induced IFN-γ, granzyme B, TNF-α and IL-10 response in humans with immunity against *L. major* and *L. infantum* infection. The capacity of *La*PSA-38S to induce mixed Th1 and Th2/T regulatory responses is an argument for the use of this protein as a potential candidate vaccine. Our approach to investigate the ability for a given antigen, to elicit cellular protective responses in human populations from different endemic areas may help to select vaccine candidates and to prioritize antigens for clinical development of a subunit vaccine against leishmaniasis.
